# *PaAIL1* Genes Modulate Floral Initiation, Floral Development, and Dormancy Regulation in *Platanus acerifolia*

**DOI:** 10.3390/genes17040393

**Published:** 2026-03-30

**Authors:** Changsheng Shao, Hui Chen, Fangfang Cai, Jiaqi Zhang

**Affiliations:** 1 Hangzhou Animation & Game College, Hangzhou Polytechnic University, Hangzhou 310018, China; 2022010018@hzvtc.edu.cn; 2 National Key Laboratory for Germplasm Innovation & Utilization of Horticultural Crops, Huazhong Agricultural University, Wuhan 430070, China; nktina1995@163.com; 3 Plant Genomics & Molecular Improvement of Colored Fiber Laboratory, College of Life Sciences and Medicine, Zhejiang Sci-Tech University, Hangzhou 310018, China

**Keywords:** AINTEGUMENTA-LIKE1, bud dormancy, floral induction, *P. acerifolia*

## Abstract

**Background/Objectives**: The coordination of flowering and dormancy represents a fundamental adaptive strategy for perennial plant survival. Recent studies have demonstrated that *AIL1* homologs act as integrators of short-day signals, playing pivotal roles in seasonal growth cessation and dormancy regulation in poplar. Despite these advances, whether AIL1-mediated regulatory mechanisms are conserved in *Platanus acerifolia*, a species with distinct phylogenetic and ecological characteristics, remains an open question. Methods: In this study, two *AIL1* homologs, *PaAIL1a* and *PaAIL1b*, were isolated from *P. acerifolia*. Their biological functions were systematically investigated through sequence analysis, spatiotemporal expression profiling, environmental stress treatments, yeast one-hybrid assays, and luciferase (LUC) transient expression assays. Results: *PaAIL1*s (*PaAIL1a* and *PaAIL1b*) exhibited ubiquitous expression across diverse tissues and organs, functioning as mediators of photoperiod and temperature signaling to orchestrate bud dormancy regulation. Interaction and activation assays placed these factors downstream of PaFUL proteins. While displaying functional redundancy in dormancy induction and floral development, the paralogs underwent distinct subfunctionalization: PaAIL1a specifically regulated flowering initiation and dormancy release, whereas PaAIL1b acted predominantly during the ecodormancy phase. Conclusions: These results establish *PaAIL1* genes as critical integrators of environmental signals and developmental programs, governing seasonal growth dynamics in this species.

## 1. Introduction

AINTEGUMENTA-LIKE (AIL) proteins are plant-specific transcription factors that belong to the APETALA2/ETHYLENE-RESPONSIVE FACTOR (AP2/ERF) superfamily; they are expressed in all plant meristems. Previous studies have demonstrated that *AIL* genes primarily regulate growth and floral-organ development, and that they are involved in the initiation of organ primordia, female gametophyte formation, organ growth, and polarity establishment, as well as in responses to abiotic stresses such as drought, freezing, and salinity [1,2].

Recent studies have revealed that AIL1 in poplar regulates cell division and proliferation, thereby promoting plant growth and development. Although SD (short-day) signals normally downregulate *AIL1* expression, this downregulation is abolished when *phyA* and *FT1* are overexpressed. In this regulatory hierarchy, *AIL1* acts downstream of the FT-FDL1 module and is targeted by Like-AP1 (LAP1) [3,4]. Transgenic plants overexpressing or silencing *AIL1* exhibited phenotypes similar to those with *LAP1* overexpression or silencing, showing delayed or accelerated responses to short-day-induced growth cessation, respectively [5,6]. Additionally, in grape (*Vitis vinifera*), *VvFT*, *VvAP1*, and *VvAIL2* showed higher expression levels in the shoot apical meristem than in lateral buds during the transition from ecodormancy to endodormancy [7]. Based on these advances in dormancy regulation research in poplar, a “short-day-induced growth cessation model” has been proposed [8,9,10]. Under long-day conditions, FT2 interacts with FDL1 to induce the expression of downstream genes *AP1* and *AIL1*; consequently, AIL1 positively regulates cell proliferation genes, ultimately enhancing shoot apical meristem (SAM) activity to promote growth. Under short-day conditions, *FT2* expression rapidly decreases, the entire pathway is suppressed, and growth cessation eventually occurs [4,11]. This photoperiodic regulatory mechanism provides a model for controlling seasonal growth in trees.

Given that *AIL1* homologs in Populus primarily function as binary switches mediating SD-induced growth cessation, we hypothesized that *AIL1* genes in *P. acerifolia*—a basal eudicot tree forming complex capitulum inflorescences—have undergone functional diversification to accommodate the antagonistic demands of prolonged floral development and seasonal dormancy. Specifically, we posit that gene duplication produced paralogs with distinct regulatory niches: one paralog (*PaAIL1a*) retained the ancestral role in growth cessation and dormancy release, while the other (*PaAIL1b*) neofunctionalized to regulate specific aspects of capitulum development and ecodormancy maintenance. This hypothesis predicts that (1) *PaAIL1* paralogs will exhibit non-redundant expression patterns across the seasonal cycle, diverging from the uniform photoperiodic response of *PtAIL1*; (2) the paralogs will differentially integrate environmental signals, with *PaAIL1a* primarily responding to SD-induced dormancy cues and *PaAIL1b* to temperature-mediated ecodormancy regulation; and (3) *PaAIL1* genes will occupy divergent positions within the floral regulatory network, potentially decoupling from the strict FT-FDL-AP1-AIL1 hierarchy characterized in Populus. Here, we test these predictions through comprehensive spatiotemporal expression analysis and functional characterization of *PaAIL1* genes.

In this study, *PaAIL1*s (*PaAIL1a*/*b*) were identified in London plane tree (*P. acerifolia*). Spatiotemporal expression analyses indicated that *PaAIL1*s are likely involved in floral induction and dormancy regulation. Yeast one-hybrid and luciferase (LUC) transient expression assays demonstrated that *PaAIL1*s are directly or indirectly activated by PaFUL1/2/3. These results reveal that *PaAIL1a*/*b* regulate floral induction and dormancy release in London plane tree.

## 2. Materials and Methods

### 2.1. Plant Material

Plant materials (*P. acerifolia*) and growth conditions for year-round and spatial expression analyses were as described previously [12]. Tobacco (Nicotiana tabacum ‘Xanthi’) was used for genetic transformation, while *Nicotiana benthamiana* (*N. benthamiana*) seedlings (3–4 weeks old) were used for subcellular localization and dual-luciferase assays. All plants were maintained in growth chambers (14 h light/10 h dark photoperiod, 25 °C).

### 2.2. RNA Extraction and Quantification

Total RNA was extracted from *P. acerifolia* samples using a modified CTAB method and subsequently used for cDNA synthesis. RNA integrity was assessed by 1% agarose gel electrophoresis, and purity was determined using a NanoPhotometer^®^ spectrophotometer (IMPLEN, Westlake Village, CA, USA). High-quality genomic DNA (gDNA) was isolated from young leaves using the standard CTAB protocol.

### 2.3. Gene Cloning and Sequence Analysis

Based on unpublished transcriptome data from *P. acerifolia*, two *AIL1* genes were identified. Gene-specific primers were designed to amplify the full-length coding sequences (cDNAs) and genomic DNA (gDNA) of *PaAIL1* (*PaAIL1a*/*b*).

Gene structure was analyzed using the Gene Structure Display Server (GSDS; http://gsds.cbi.pku.edu.cn/ (accessed on 25 March 2026)), conserved domains were identified using SMART (http://smart.embl-heidelberg.de/ (accessed on 25 March 2026)), multiple sequence alignment was performed using GeneDoc (http://nrbsc.org/gfx/genedoc/ (accessed on 25 March 2026)), and phylogenetic analysis was conducted using MEGA 11 with the Maximum Likelihood (ML) method (1000 bootstrap replicates). All primer sequences are listed in Supplementary Table S1.

Promoter regions of *PaAIL1* (*PaAIL1a*/*b*) were cloned using fusion primer and nested integrated PCR (FPNI-PCR) [13]. Transcription start sites and putative cis-regulatory elements were predicted using PLACE) (https://www.dna.affrc.go.jp/PLACE/?action=newplace (accessed on 25 March 2026)) and PlantCARE (http://bioinformatics.psb.ugent.be/webtools/plantcare/html/ (accessed on 25 March 2026)).

### 2.4. Quantitative Real-Time PCR

For expression analysis, quantitative real-time PCR (qRT-PCR) was conducted using *PaTPI* (*P. acerifolia* triose phosphate isomerase) as the endogenous reference gene. The 2^−ΔΔCT^ method was used to determine the relative expression levels of the genes. Data are presented as the mean values ± SD (standard deviation) of three replicates. The primers used in this work are listed in Table S1. All analyses included three biological replicates (Lines 1–3), with each sample run in triplicate (three technical replicates).

The total RNA extracted from *P. acerifolia* samples and Prime Script reverse transcriptase (RT) (Takara, Otsu, Japan) were used to synthesize the cDNAs that were used as templates for quantitative real-time PCR (qRT-PCR). SYBR Premix Ex Taq (Takara, Okinawa, Japan) and ABI 7500 Real-Time System (Applied Biosystems, Waltham, MA, USA) were used to perform qRT PCRs. qRT-PCR amplifications were performed in a 10 μL volume reaction, containing 1.0 μL template, 5.0 μL 2 × SYBR Green Master Mix, 0.2 μL forward and reverse primers (10 mL mol/mL), and 3.6 μL ddH_2_O.

### 2.5. Photoperiod/Temperature Treatments

Experimental treatments comprising low temperature (LT, 8 °C), a short photoperiod (SD, 8L:16D), and combined LT/SD were established according to [12]. Prior to treatment imposition, all specimens were acclimatized in a growth chamber under control conditions (25 °C, 14 h light/10 h dark).

### 2.6. Yeast One-Hybrid Assay and Dual-Luciferase Assay

The promoters of *PaAIL1* genes were divided into four overlapping fragments (~500 bp each, overlapping adjacent fragments by ~50 bp). These fragments were individually cloned into pHIS2.1 (for yeast one-hybrid assays) and pBGWL7 (for dual-luciferase assays) to generate reporter constructs. Effector constructs were generated using *PaFUL1* (KY432367), *PaFUL2* (KY432368), and *PaFUL3* (GU296505), which were cloned into pGADT7 and pCAMBIA2300s, respectively.

For yeast one-hybrid (Y1H) assays, reporter and effector vectors were co-transformed into yeast strain Y187. Transformants were selected on SD/-Leu/-Trp medium and subsequently assayed on SD/-Leu/-Trp/-His medium supplemented with an appropriate concentration of 3-amino-1,2,4-triazole (3-AT).

For dual-luciferase assays, reporter constructs (pBGWL7-promoter) were introduced into Agrobacterium tumefaciens GV3101 containing the helper plasmid pSoup. Effector constructs (pCAMBIA2300s-*PaFUL*) were separately transformed into GV3101. Agrobacterium cultures harboring reporter and effector constructs were co-infiltrated into the abaxial surface of N. benthamiana leaves. Luciferase activity was measured using the NightShade LB 985 In Vivo Plant Imaging System (Berthold, Bad Wildbad, Germany).

### 2.7. Statistical Treatment

Statistical significance was assessed by one-way analysis of variance (ANOVA) using SPSS software (IBM SPSS statistics 27), with *p* < 0.05 considered statistically significant. All data are presented as mean ± standard deviation (SD) from three independent biological replicates. Each experimental procedure was independently performed in triplicate.

## 3. Results

### 3.1. Molecular Cloning and Structural Characterization of AIL1 in P. acerifolia

Previous transcriptome analysis identified differentially expressed *AIL* genes during *P. acerifolia* dormancy transitions, suggesting roles in seasonal growth regulation. Here, we characterized two *AIL1* homologs, *PaAIL1a* and *PaAIL1b* (GenBank: PX990732/PX990733), denoted collectively as *PaAIL1a*/*b*.

**Figure 1:**
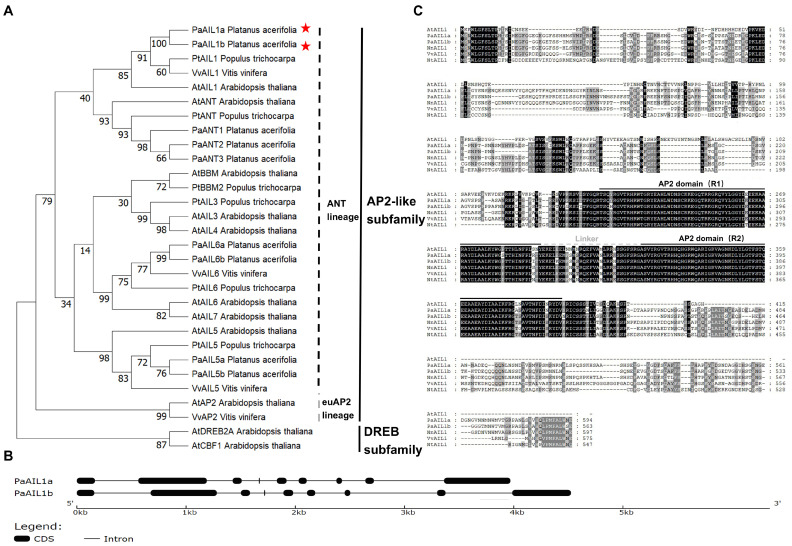
The sequence alignment and phylogenetic tree analysis of PaAIL1a/b. (**A**) The phylogenetic tree of PaAIL1a/b and AP2 family proteins from other species. The genes which are marked by a red pentacle are PaAIL1a/b. Numbers at nodes indicate bootstrap support values based on 1000 replicates. The evolutionary history was inferred using the Maximum Likelihood method and JTT matrix-based model. (**B**) The structure of *PaAIL1a*/*b*. Gene structures were analyzed and visualized using the Gene Structure Display Server (http://gsds.cbi.pku.edu.cn/ (accessed on 25 March 2026)); black boxes indicate exons, and horizontal lines indicate introns. (**C**) The conserved domains of PaAIL1a/b proteins. The multiple sequence alignment was performed and visualized using GeneDoc, and the conserved domains were analyzed using SMART (http://smart.embl-heidelberg.de/ (accessed on 25 March 2026)). The AP2 domains are indicated by black solid lines under sequences, the linker region is indicated by the gray dotted line.

Phylogenetic reconstruction placed PaAIL1 proteins within the AINTEGUMENTA (ANT) clade, specifically clustering with *Arabidopsis* AIL1 in a distinct sub-clade comprising AIL1 and ANT proteins (Figure 1A).

Structural analysis revealed conserved gene architecture: both genes contain eight exons and seven introns (Figure 1B), with coding sequences of 1782 bp (*PaAIL1a*) and 1689 bp (*PaAIL1b*) encoding 594-aa and 563-aa proteins, respectively. Sequence alignment identified characteristic AP2 domains—R1 (73 aa, N-terminal) and R2 (65 aa, C-terminal)—connected by a linker region (Figure 1C), consistent with AIL protein topology. These features confirm PaAIL1a/b as ANT lineage transcription factors within the ERF/AP2 superfamily.

### 3.2. Subcellular Localization of PaAIL1a/b

**Figure 2:**
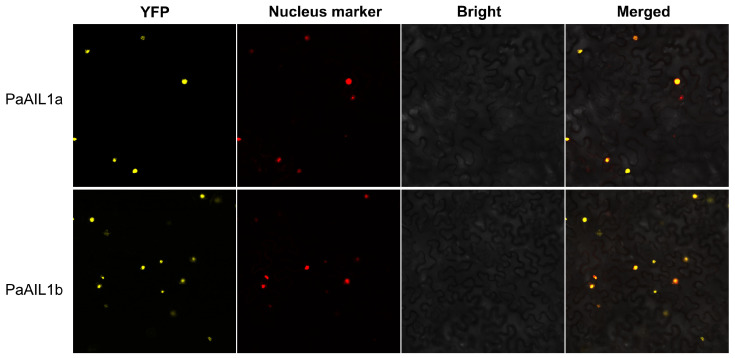
Subcellular localization of PaAIL1a/b protein in tobacco leaves. 35S:YFP is an empty vector control. The red fluorescent part indicates the nucleus. Bright: bright field image; YFP: YFP fluorescence; Nucleus marker: histone H2B-RFP fluorescence; Merged: merged images of Bright, YFP and RFP fields. Scale bars 50 mm. RFP, red fluorescent protein.

To further pinpoint the functional sites of PaAIL1a/b, we determined their subcellular localization. Both proteins were found exclusively in the nucleus (Figure 2), consistent with the expected behavior of transcription factors that operate within this compartment.

### 3.3. Spatiotemporal Expression Patterns of PaAIL1a/b in P. acerifolia

**Figure 3:**
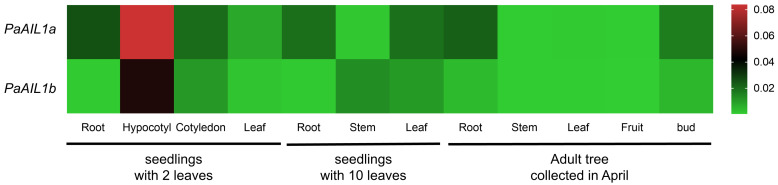
Spatiotemporal expression profiling of *PaAIL1a*/*b* across developmental gradients. Tissue samples were harvested from three distinct ontogenetic stages: early seedlings (cotyledon stage, 2 true leaves), juvenile saplings (1-year-old, 10-leaf stage), and mature arboreal tissues (40-year-old specimens). Transcript abundance was quantified via quantitative real-time PCR, with PaTPI serving as the endogenous reference gene for normalization.

To characterize the spatiotemporal expression patterns of *PaAIL1a*/*b*, quantitative real-time PCR (qRT-PCR) was performed across various tissues and developmental stages of *P. acerifolia*. Transcripts of both genes were detected ubiquitously, though with distinct tissue-specific profiles (Figure 3). Both exhibited peak expression in the hypocotyls of two-leaf seedlings; however, *PaAIL1a* maintained substantial expression in roots across developmental stages, while *PaAIL1b* showed relatively weak expression in non-hypocotyl tissues. These results suggest partial functional redundancy accompanied by subfunctionalization between the paralogs.

**Figure 4:**
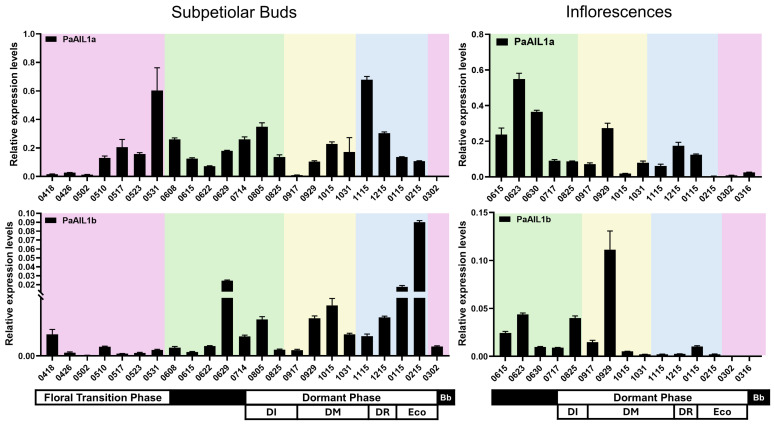
Seasonal transcript dynamics of *PaAIL1a*/*b* in subpetiolar buds and floral primordia of *P. acerifolia*. Samples were collected from mature field-grown specimens in Wuhan, Hubei Province, China (113°41′–115°05′ E, 29°58′–31°22′ N) across phenological cycles. The *x*-axis displays monthly sampling dates chromatically categorized by seasonal phases, with spring (red), summer (green), autumn (yellow), and winter (blue) denoted by distinct colors. Based on bud burst indices coupled with endogenous hormone profiles, the dormancy continuum was classified into paradormancy induction (DI), endodormancy maintenance (DM), dormancy release (DR), and ecodormancy (Eco) stages. Transcript abundance was quantified via qRT-PCR using *TPI* as the internal calibrator; data represent means ± standard deviation.

We further examined annual expression cycles of *PaAIL1a*/*b* in subpetiolar buds collected periodically from April through March. qRT-PCR revealed distinct seasonal expression patterns between the paralogs (Figure 4). *PaAIL1a* exhibited three major expression waves: (i) May to mid-June (floral induction and inflorescence differentiation), (ii) late June to late August (dormancy induction), and (iii) late September to mid-February (dormancy maintenance), with basal levels during intervening periods. By contrast, *PaAIL1b* displayed four distinct peaks: late June (inflorescence differentiation), mid-July to early September (dormancy induction), late September to mid-October (dormancy maintenance), and mid-January to mid-February (dormancy release and ecodormancy).

During floral induction, *PaAIL1a* expression increased in May, peaked at the end of May/early June, and remained elevated through subsequent stages. *PaAIL1b*, however, peaked in mid-May, declined, then resurged sharply during single-flower differentiation in late June (Figure 4). These divergent patterns suggest functional specialization: *PaAIL1a* appears to regulate both floral induction and subsequent growth maintenance, whereas *PaAIL1b* functions primarily during early induction and single-flower differentiation.

Throughout dormancy induction (July–November), *PaAIL1a* maintained high expression levels (peaking in early August), while *PaAIL1b* remained relatively low yet followed a similar expression trajectory (Figure 4). This indicates functional redundancy during dormancy establishment, with *PaAIL1a* acting throughout the entire induction period and *PaAIL1b* contributing primarily during early phases. During dormancy maintenance and release, expression patterns diverged markedly. *PaAIL1a* increased in late September (early maintenance), peaked in late November (early release), and remained elevated through February (release and ecodormancy). *PaAIL1b*, however, peaked in mid-October (maintenance), declined, then resurged in December, reaching maximum levels in mid-February (ecodormancy) before decreasing sharply (Figure 4). The elevated expression of both genes during dormancy release and ecodormancy suggests cooperative roles in dormancy termination.

Expression profiling throughout main inflorescence development revealed three distinct upregulation events for both genes: the first occurred from mid-June to late June (floral meristem differentiation), the second from mid-September to late September, and the third from mid-November to mid-January of the following year (inflorescence developmental arrest) (Figure 4) [12]. Notably, expression patterns in inflorescences closely paralleled those observed in axillary buds. Collectively, these findings demonstrate that PaAIL1a/b play multifaceted, partially redundant yet distinct roles in floral initiation, inflorescence development, and seasonal dormancy cycling.

### 3.4. PaAIL1a/b Expression Under LD, LT, and SD Treatments

**Figure 5:**
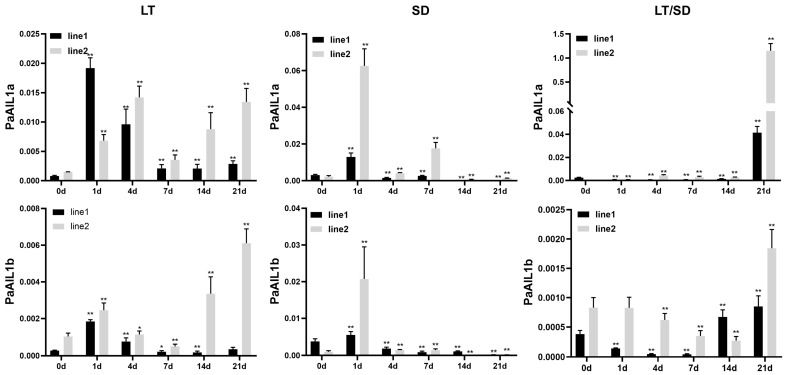
The expression of *PaAIL1a*/*b* genes in leaves under short-day and low-temperature conditions. Gene expression kinetics were monitored under three experimental conditions: low-temperature (LT, left panels), short-day (SD, middle panels), and combined SD + LT stress (right panels). Leaf tissues were excised from 1-year-old juvenile specimens at 4.5 h post-illumination. Statistical significance relative to control samples (0 d) was assessed by univariate analysis of variance, with * *p* < 0.05 and ** *p* < 0.01 denoting differential significance levels. The methodological specifics follow the protocols established in Figure 4.

As noted above, *PaAIL1a*/*b* exhibited markedly elevated expression during dormancy release and ecodormancy—stages regulated by photoperiod and temperature cues. To dissect these environmental responses, we analyzed *PaAIL1a*/*b* expression under low-temperature (LT), short-day (SD), and combined LT/SD conditions (Figure 5). During the initial 0–7 day period, neither SD nor LT alone induced consistent expression patterns. However, by days 14 and 21, divergent responses emerged: *PaAIL1a*/*b* were significantly upregulated under LT but downregulated under SD conditions.

Under combined LT/SD conditions, *PaAIL1a* expression gradually increased during days 1–7, rose sharply at day 14, and reached peak levels hundreds-fold higher than the untreated controls (day 0) by day 21. In contrast, *PaAIL1b* showed no discernible pattern during days 1–7 but exhibited a sharp increase at day 21 (Figure 5). Notably, the response profiles of *PaAIL1a*/*b* to LT and LT/SD closely paralleled those of *PaFT*, a key dormancy regulator in *P. acerifolia* [14]. These results indicate that *PaAIL1a*/*b* are repressed by prolonged SD signals yet induced by sustained LT and combined LT/SD conditions, with *PaAIL1a* potentially playing a predominant role in this environmental sensing mechanism.

### 3.5. Interactions Between FUL Proteins and PaAIL1a/b Promoters

**Figure 6:**
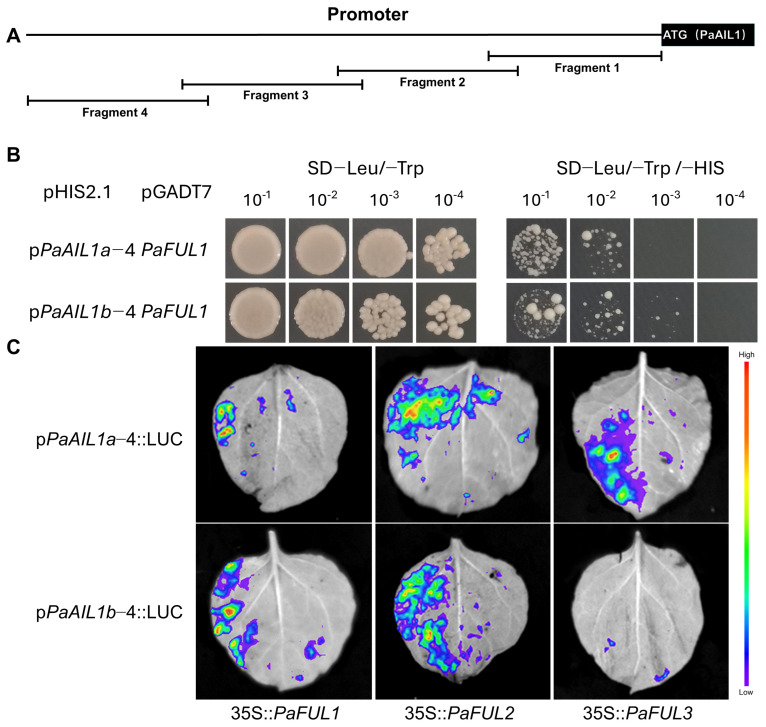
The interactions between PaFUL proteins and *PaAIL1a*/*b* promoters. (**A**) Architectural organization of *PaAIL1a*/*b* proximal promoters, illustrating the segmental deletions used for interaction mapping. (**B**) Y1H (yeast one-hybrid) screening demonstrating direct binding of PaFUL1 to distinct promoter fragments. (**C**) Transient transactivation assays in planta via dual-luciferase re-porter systems; firefly luciferase activity driven by *PaAIL1a*/*b* promoters was normalized against Renilla luciferase, with empty pCAMBIA2300s vectors serving as negative controls.

Given the positive correlation between *PaFUL* and *PaAIL1a*/*b* expression levels observed previously [15], we conducted yeast one-hybrid (Y1H) assays to determine whether PaFUL proteins directly interact with *PaAIL1a*/*b* promoters. Self-activation tests confirmed that *PaAIL1a*/*b* promoter fragments were effectively suppressed by supplementation with 3-amino-1,2,4-triazole (3-AT) (Figure 6A,B and Figure S1). Subsequent interaction assays revealed that PaFUL1 specifically bound to both *PaAIL1a* and *PaAIL1b* promoters in yeast, whereas PaFUL2 and PaFUL3 showed no detectable binding to either promoter (Figure 6B).

To further verify the interaction between FUL proteins and the *PaAIL1a*/*b* promoters, we performed a dual-luciferase assay in vivo. As shown in Figure 6C, unlike the yeast assay, PaFUL1/2/3 could trigger a fluorescent signal by interacting with the *PaAIL1a* promoter in vivo; when interacting with the *PaAIL1b* promoter in vivo, all PaFULs except PaFUL3 could trigger the fluorescent signal. These results demonstrate that PaFUL1 physically binds to both *PaAIL1a* and *PaAIL1b* promoters (Y1H) and activates their transcription. Notably, while PaFUL2 and PaFUL3 showed no detectable DNA binding in yeast, they were capable of activating *PaAIL1a* promoter-driven expression in planta. This discrepancy suggests that PaFUL2/3 may regulate *PaAIL1* expression indirectly or require plant-specific co-factors/modifications absent in yeast cells. PaFUL3, however, failed to activate the *PaAIL1b* promoter in both assays, indicating paralog-specific target preferences.

## 4. Discussion

Spatiotemporal expression analysis revealed that *PaAIL1a* exhibited high expression in the roots of *P. acerifolia* at different developmental stages, while *PaAIL1b* transcripts were barely detectable (Figure 3). Previous studies have demonstrated that *AINTEGUMENTA-LIKE1* (*PtAIL1*) controls adventitious root primordia formation in Populus [16], suggesting that the two *AIL1* paralogs in *P. acerifolia* have undergone functional divergence, with *PaAIL1a* retaining the conserved function. Previous studies have also shown that hypocotyl elongation is strongly influenced by light and phytohormones [17]. The high expression of *PaAIL1a*/*b* in hypocotyls suggests that these genes may also be regulated by light or hormones.

Annual expression analysis showed that *PaAIL1a* was highly expressed in the subpetiolar buds during the floral induction (Figure 4), while *PaAIL1b* showed moderate expression in early floral induction and peaked again during single-flower differentiation (Figure 4). Yeast one-hybrid assays demonstrated that PaFUL1 directly binds to both *PaAIL1a*/*b* promoters, while dual-luciferase (LUC) reporter assays indicated that *PaAIL1a* can be activated by PaFUL1/2/3 in planta (Figure 6). Notably, given that PaFUL2/3 showed no binding in Y1H assays, their activation of *PaAIL1a* likely occurs through indirect mechanisms or requires additional plant-specific factors [15]. Collectively, these findings indicate that *PaAIL1a* and *PaAIL1b* contribute to floral induction regulation in *P. acerifolia* but have undergone significant functional divergence: *PaAIL1a* is primarily involved in the initial floral induction phase and maintains functional roles during single-flower differentiation, whereas *PaAIL1b* predominantly participates in single-flower differentiation within the capitulum while potentially contributing to early floral induction.

Decades of research have established a four-phase classification of bud dormancy in perennial plants: induction, endodormancy, release, and ecodormancy [11,18,19,20]. Consistent with this framework, *P. acerifolia* exhibits this four-phase dormancy pattern, with *PaAIL1a*/*b* showing phase-specific expression profiles (Figure 4). During dormancy induction, *PaAIL1a*/*b* expression initially increased then declined as day length shortened; during dormancy establishment and maintenance, *PaAIL1a* gradually increased while *PaAIL1b* remained low. Thereafter, the *PaAIL1a* gene was upregulated at the later stage of dormancy maintenance and peaked in the dormancy release phase, while *PaAIL1b* began to be upregulated at the late stage of dormancy release and peaked in the ecodormancy phase. *PaAIL1a*/*b* expression tracked with known dormancy regulators in a phase-dependent manner: both paralleled *PaFTL* and *PaFUL1*/*2*/*3* during induction, but subsequently diverged—*PaAIL1a* aligned with *PaFT*/*PaFUL2* during release, while *PaAIL1b* associated with *PaFUL1*/*PaFTL* during ecodormancy [14,15]. These temporal correlations, coupled with the critical roles of *FT* and *AP1*/*FUL-like* genes in dormancy [15,21,22,23], indicate that *PaAIL1a*/*b* likely modulate *P. acerifolia* dormancy, acting redundantly during induction but specializing in later phases.

Dormancy induction is primarily triggered by short-day (SD) photoperiodic signals, whereas chilling serves as the main cue for dormancy release in *P. acerifolia* [24,25]. Comparative analysis with poplar reveals that *AIL1* functions as a critical molecular switch that connects photoperiodic signal perception with meristem activity maintenance. During the growing season, the FT2-LAP1-AIL1 pathway activates the cell cycle to sustain shoot apical meristem growth; however, under autumn SD conditions, *AIL1* suppression leads to cell cycle arrest, thereby inducing dormancy [5,18]. Similarly, *PaFTL* expression is downregulated by prolonged SD signals and may participate in regulating SD-mediated dormancy induction in *P. acerifolia* [12]. Analogous to the expression pattern of *LAP1* in poplar, *PaFUL* expression in *P. acerifolia* is also suppressed by extended SD conditions, with *PaFUL2*/*3*, especially *PaFUL2*, playing roles in dormancy regulation [15]. Our experimental results demonstrate that *PaAIL1a*/*b* genes exhibit expression trends consistent with *PaFTL* under SD conditions (Figure 5), and *PaAIL1a*/*b* expression can be activated by *PaFUL2* (Figure 6). These findings suggest the existence of a dormancy induction pathway in *P. acerifolia* analogous to that in poplar, namely the PaFTL-PaFUL2-PaAIL1 axis.

Under LT and combined LT/SD treatments, *PaAIL1a*/*b* displayed response patterns congruent with those of *PaFT* and *PaFUL2*/*3* (Figure 5). Notably, *PaFT* expression is upregulated under individual LT conditions and shows even stronger induction under combined LT/SD treatment, indicating that this gene specifically mediates LT-induced dormancy release [12]. Furthermore, under LT/SD treatment, *PaFUL2*/*3* expression exhibited an upward trend consistent with *PaFT* [15]. Combined with yeast one-hybrid and LUC activation assays (Figure 6), these results suggest a putative regulatory axis involving PaFT-PaFUL2/3-PaAIL1. However, the absence of detectable binding between PaFUL2/3 and *PaAIL1* promoters in Y1H assays indicates that this regulation may be indirect, or that PaFUL2/3 function as part of a protein complex rather than as direct DNA-binding regulators in this context. Additionally, annual expression profiling in subpetiolar buds revealed distinct temporal patterns: *PaAIL1a* exhibited higher expression levels during the dormancy release stage, whereas *PaAIL1b* expression increased during the ecodormancy stage (Figure 4). This differential expression suggests that *PaAIL1a* may play a more prominent role during dormancy release in *P. acerifolia*.

The coordinated regulation of flowering and dormancy by *PaAIL1* genes reveals a sophisticated signal integration mechanism that converges photoperiodic and temperature cues. Our data support a model in which *PaAIL1* genes function as molecular rheostats downstream of the PaFT-PaFUL signaling hierarchy, with PaFUL1 serving as a direct transcriptional activator of *PaAIL1* promoters, whereas PaFUL2/3 likely regulate *PaAIL1* expression indirectly or require plant-specific cofactors for effective DNA binding (Figure 6). Under short-day (SD) conditions, the downregulation of *PaFT* leads to reduced *PaFUL2*/*3* expression, subsequently alleviating the activation of *PaAIL1* genes, thereby simultaneously arresting floral meristem activity and inducing dormancy. Conversely, under low-temperature (LT) or combined LT/SD conditions, *PaAIL1* expression is upregulated (Figure 4 and Figure 5), potentially through a chilling-mediated epigenetic mechanism or via alternative regulatory pathways that override SD repression. This dual responsiveness positions PaAIL1 as a critical decision-making node: high PaAIL1 activity promotes cell proliferation for both floral organogenesis and bud break, whereas reduced activity induces growth cessation. Notably, the distinct response kinetics of the two paralogs—*PaAIL1a* showed rapid induction under combined LT/SD stress, while *PaAIL1b* responded more slowly—suggest temporal partitioning of signal integration, allowing fine-tuned adaptation to gradual seasonal transitions.

Our findings challenge the traditional view that floral induction and dormancy establishment are discrete developmental stages. Instead, *PaAIL1* expression patterns suggest that these processes represent a developmental continuum orchestrated by shared regulatory modules. During the floral induction phase (May–June), elevated *PaAIL1a* expression supports meristematic proliferation necessary for inflorescence differentiation, while later in the season (July–August), sustained *PaAIL1* activity in subpetiolar buds prepares the meristem for dormancy entry by gradually downregulating cell cycle genes.

This ‘preparation-for-dormancy’ hypothesis implies that the same meristematic events required for floral organogenesis (cell division, differentiation) must be actively suppressed during dormancy onset. *PaAIL1* genes appear to mediate this transition by shifting from promoting floral primordia growth to maintaining meristem potential during endodormancy. The functional bifurcation of the paralogs supports this model: *PaAIL1a* maintains basal activity throughout dormancy to preserve meristem identity, while *PaAIL1b* specifically activates during ecodormancy to prime floral development upon spring awakening. This regulatory architecture ensures that floral initiation and dormancy are antagonistic yet functionally linked processes.

Comparative analysis reveals both conserved regulatory architectures and species-specific innovations in AIL1-mediated seasonal growth regulation. In Populus, PtAIL1 functions as a binary switch responding primarily to photoperiod via the FT-LAP1-AIL1 cascade, with AIL1 directly controlling cell cycle exit during SD-induced growth cessation [4,5]. Similarly, in Platanus, we identified a PaFTL-PaFUL2-PaAIL1 axis that mediates SD-induced dormancy induction, suggesting deep conservation of this regulatory module across Salicaceae and Platanaceae.

However, significant divergences indicate adaptive evolution in response to distinct ecological niches. Unlike Populus, where a single *AIL1* gene predominates, *P. acerifolia* has evolved functionally divergent paralogs with distinct temporal niches: PaAIL1a regulates both flowering initiation and dormancy release, while *PaAIL1b* specializes in single-flower differentiation and ecodormancy maintenance. This subfunctionalization may reflect the distinct reproductive strategy of Platanus, which forms complex capitulum inflorescences requiring prolonged meristematic activity compared to the catkins of Populus.

Furthermore, in grape (*V. vinifera*), *VvAIL2* shows higher expression during ecodormancy-to-endodormancy transitions [7], resembling PaAIL1b expression patterns, suggesting that *AIL* gene family expansion and neofunctionalization may be a general evolutionary response to complex seasonal cycles in long-lived perennials. These comparative insights position *PaAIL1* genes as evolutionary ‘tuning knobs’ that modulate the trade-off between reproductive investment and stress survival across diverse woody lineages.

## 5. Conclusions

In conclusion, *PaAIL1a*/*b* are direct downstream targets of PaFUL1, and may respond to PaFUL2/3-mediated signals, likely through indirect mechanisms, responding to short-day (SD) signals to regulate dormancy induction. Specifically, *PaAIL1a* may mediate dormancy release, whereas *PaAIL1b* may function during subsequent ecodormancy. These findings establish *PaAIL1* genes as pivotal integrators coupling reproductive development with seasonal dormancy, providing a mechanistic basis for the long-observed but poorly understood coordination between flowering time and winter survival in temperate trees.

## Data Availability

The data supporting the results are available online in the paper and in the Supplementary Materials.

## Supplementary Materials

The following supporting information can be downloaded at: https://www.mdpi.com/article/10.3390/genes17040393/s1, Figure S1: Self-activation tests of *PaAIL1a*/*b* promoter fragments; Table S1: Primers used in this study.

## Abbreviations

The following abbreviations are used in this manuscript:

LT	Low temperature
SD	Short day
3-AT	3-amino1,2,4-triazole
