# Supplementary material for: PaAIL1 Genes Modulate Floral Initiation, Floral Development, and Dormancy Regulation in Platanus acerifolia
Source: Genes (Basel). 2026 Mar 30;17(4):393. doi: 10.3390/genes17040393 (PMC13116398; doi:10.3390/genes17040393)
Supplement: Supplementary file 1 [file genes-17-00393-s001.zip › genes-4220491-supplementary.pdf]

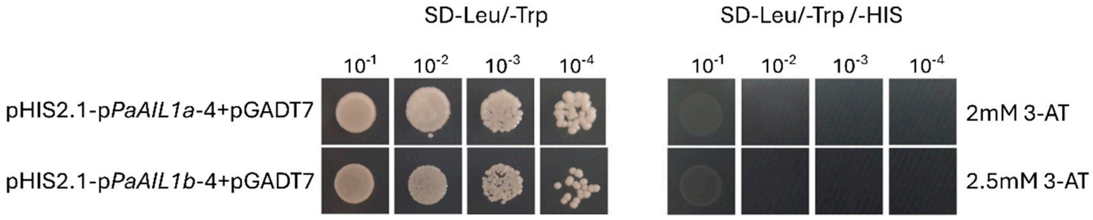

Figure S1. Self-activation tests of *PaAIL1a*/*b* promoter fragments.

Table S1. Primers used in this study

Primer	Sequence (5'- 3')
PaAIL1a-F	AAAAGAAGAGTAGAAAATGAGTAAC
PaAIL1a-R	TGCAACCACGTTATGAAACAATAGT
PaAIL1b-F	AGATGATCGGTTGCCTGGGTTTTTC
PaAIL1b-R	TTGACGTTCATGTGGAATTTATTTC
qRT-PaAIL1a- F	CACTCTGCAGCATCACACAGTGAGT
qRT-PaAIL1a-R	TAACGCCATCTCTCAAGCTTCTTAC
qRT-PaAIL1b-F	TTCCAGTGACTCGGTGTCGCAG
qRT-PaAIL1b-R	ACCTCTCCATACTCTCACTCACTCC
pPaAIL1a-F	CCAACCCGAGCGTGAATAAATA
pPaAIL1a-R	GTGAGGTTTATGGTGAAAAATGGA
pPaAIL1b-F	CCTCTGCTTGTGTGATGAACCTG
pPaAIL1b-R	CCTTCGTTGATACTCAGGTGTGGC
AD-FUL1-F	gtaccagattacgctcatatgATGGGGAGAGGTAGGGTTCAA
AD-FUL1-R	actggcctccatggccatatgCTATAAATTCACGTGACGAAGCATC
AD-FUL2-F	gtaccagattacgctcatatgATGGGGAGAGGTAGGGTTCAGC
AD-FUL2-R	actggcctccatggccatatgCTATTCAGTCACGTGACTAAGCATCC
AD-FUL3-F	gtaccagattacgctcatatgATGGGGAGAGGTAGGGTTCTGT
AD-FUL3-R	actggcctccatggccatatgCTATTCATTTACATGACGAAGCATCC
PaAIL1a-PRO-1F	ctatagggcgaattcgagctcAGGACGAACGAAGTCCGAGG
PaAIL1a-PRO-1R	agtggatccacgcgtgagctcCATTTTCTACTCTTCTTTTTTTTTTTCG
PaAIL1a-PRO-2F	ctatagggcgaattcgagctcGTTTTGTATAAGAGACCCGGTCTTTA
PaAIL1a-PRO-2R	agtggatccacgcgtgagctcTTGGATTCTCGTTTCATTTTCTCA
PaAIL1a-PRO-3F	ctatagggcgaattcgagctcAAATTTGATTCGTGCACCTACATT
PaAIL1a-PRO-3R	agtggatccacgcgtgagctcAATTTTTTATATCTCAGACTCTCATTCTGC
PaAIL1a-PRO-4F	ctatagggcgaattcgagctcCCAACCCGAGCGTGAATAAA
PaAIL1a-PRO-4R	agtggatccacgcgtgagctcTAATCTGACAATTTGATACATTTAATCACA
PaAIL1b-PRO-1F	ctatagggcgaattcgagctcCTTTTTTGTGATTTTTGAAAACTGTTT
PaAIL1b-PRO-1R	agtggatccacgcgtgagctcCATATTCTACATAAACTCAAATATCAAACTTC

PaAIL1b-PRO-2F	ctatagggcgaattcgagctcGGATTTCGCAAAAGGTCCTTG
PaAIL1b-PRO-2R	agtggatccacgcgtgagctcCCCCCGTATCCTCATACACAAA
PaAIL1b-PRO-3F	ctatagggcgaattcgagctcTAGAAGCTTGAAACTGTAAACTATCAAAA
PaAIL1b-PRO-3R	agtggatccacgcgtgagctcTCATTGGATTGAGTTGAGTCTCAGA
PaAIL1b-PRO-4F	ctatagggcgaattcgagctcCCTCTGCTTGTGTGATGAACCTG
PaAIL1b-PRO-4R	agtggatccacgcgtgagctcCGGTTTTCATTGATTTTGACCA
v97-PaAIL1a-4F	CCAACCCGAGCGTGAATAAATAAAA
v97-PaAIL1a-4R	<u>CTCGAG</u>TAATCTGACAATTTGATACATTTAA
v97-PaAIL1b-4F	CCTCTGCTTGTGTGATGAACCTGAG
v97-PaAIL1b-4R	<u>CTCGAG</u>CGGTTTTCATTGATTTTGACCAGTT
